# Influence of longitudinal radiation exposure from microcomputed tomography scanning on skeletal muscle function and metabolic activity in female CD‐1 mice

**DOI:** 10.14814/phy2.13338

**Published:** 2017-07-04

**Authors:** John S. Mikhaeil, Sandra M. Sacco, Caitlin Saint, William Gittings, Jordan Bunda, Cameron R. Giles, Val Andrew Fajardo, Rene Vandenboom, Wendy E. Ward, Paul J. LeBlanc

**Affiliations:** ^1^ Centre for Bone and Muscle Health Brock University St. Catharines Ontaria Canada; ^2^ Department of Health Sciences Faculty of Applied Health Sciences Brock University St. Catharines Ontaria Canada; ^3^ Department of Kinesiology Faculty of Applied Health Sciences Brock University St. Catharines Ontaria Canada

**Keywords:** Citrate synthase, extensor digitorum longus, soleus, X‐ray

## Abstract

Microcomputed tomography (*μ* CT) is an imaging technology to assess bone microarchitecture, a determinant of bone strength. When measured in vivo, *μ* CT exposes the skeletal site of interest to a dose of radiation, in addition to nearby skeletal muscles as well. Therefore, the aim of this study was to determine the effects of repeated radiation exposure from in vivo *μ* CT on muscle health – specifically, muscle morphometrics, contractile function, and enzyme activity. This study exposed the right hind limb of female mice to either a low (26 cGy) or moderate (46 cGy) dose, at 2, 4, and 6 months of age, while the left hind limb of the same animal was exposed to a single dose at 6 months to serve as a nonirradiated control. Muscle weight, cross‐sectional area, isometric contractile function, and representative maximal enzyme activities of amino acid, fatty acid, glucose, and oxidative metabolism in extensor digitorum longus (EDL) and soleus were assessed. Low‐dose radiation had no effect. In contrast, moderate‐dose radiation resulted in a 5% increase in time‐to‐peak tension and 16% increase in half‐relaxation time of isometric twitches in EDL, although these changes were not seen when normalized to force. Moderate‐dose radiation also resulted in an ~33% decrease in citrate synthase activity in soleus but not EDL, with no changes to the other enzymes measured. Thus, three low doses of radiation over 6 months had no effect on contractile function or metabolic enzyme activity in soleus and EDL of female mice. In contrast, three moderate doses of radiation over 6 months induced some effects on metabolic enzyme activity in soleus but not EDL. Future studies that wish to investigate muscle tissue that is adjacent to scanned bone should take radiation exposure dose into consideration.

## Introduction

In vivo microcomputed tomography (*μ*CT) is a three‐dimensional imaging technology used to provide a longitudinal assessment of bone structure in mice (Sacco et al. 2017) and rats (Longo et al. 2017). This technology lends for a more powerful study design because it minimizes the number of animals required for a study. Although, repeated in vivo *μ*CT scanning is a powerful tool for visualizing and quantifying three‐dimensional microstructure and geometry of bone tissue, the ability for repeated X‐ray radiation exposure to alter bone health and structure has been a concern. Previous research applying moderately high doses of radiation (77–85 cGy) over shorter periods of time (3–4 weeks) with relatively shorter scanning intervals (1–2 weeks) has demonstrated impairments in trabecular structure in male and female mice (Klinck et al. 2008; Laperre et al. 2011). In contrast, recent work from our laboratory has shown that longitudinal assessment of bone structure in male and female mice at low to moderate doses of radiation (26–46 cGy) over longer periods of time (4 months) with longer scanning intervals (2 months) does not affect bone structure (Sacco et al. 2017). Thus, low to moderate radiation doses at longer intervals and over a longer period provide a reasonable image quality of the bone microarchitecture without affecting the structural properties of the bone tissue under examination.

There is a close functional relationship between bone and muscle. This is accomplished through endocrine crosstalk at both a mechanical and biochemical level (Brotto and Bonewald 2015). Thus, if muscle strength or function is altered, this could in turn impact bone health. In addition, given its proximity to bone, it is currently unknown if repeated exposures to low or moderate X‐ray radiation doses due to in vivo *μ*CT scanning would affect skeletal muscle. Previous research has examined high to very high X‐ray doses (120–1600 cGy) on rodent skeletal muscle, demonstrating no changes in muscle weight (Chowdhury et al. 2016), decreased mitochondrial DNA transcription (Gubina et al. 2010), decreased cross‐sectional area and increased centralized nuclei (Hardee et al. 2014). The only study to examine an X‐ray dose similar to this study looked at markers of mouse‐mixed hind limb protein synthesis and degradation after a single dose of 50 cGy, demonstrating increased muscle turnover (Fix et al. 2016). To date, no study has examined X‐ray doses used for assessment of bone structure by *μ*CT repeatedly over time on functional aspects of skeletal muscle – specifically in vitro muscle function and metabolic activity.

This study is novel as it shifts the focus away from bone structure to examine the effects of radiation from repeated in vivo *μ*CT on skeletal muscle. When assessing bone structure using in vivo *μ*CT, it is inevitable that the neighboring muscle will be exposed to radiation, and so for studies that wish to examine the bone‐muscle unit, it is important to understand if there are any effects due to radiation on skeletal muscle function and metabolic activity. This study is part of our previously published study examining the influence of in vivo *μ*CT on bone microstructure (Sacco et al. 2017). Female mice were selected to examine skeletal muscle size, function, and metabolic activity as previous work has shown that nutritional programming effects on bone development are seen in females and not males (Kaludjerovic and Ward 2013, 2015; Ward et al. 2016). Thus, the purpose of this study was to determine the effects of exposure to low (26 cGy) and moderate (46 cGy) radiation doses (doses that provide acceptable bone image quality) at 2, 4, and 6 months of age on muscle morphometrics, contractile function, and metabolic activity of fast‐twitch glycolytic (extensor digitorum longus; EDL) and slow‐twitch oxidative (soleus) skeletal muscles. It was hypothesized that three scans of low or moderate radiation in the same mouse would not result in significant changes in skeletal muscle morphometrics, contractile function, or metabolic activity.

## Materials and Methods

### Animals and diets

This study represents a subset of a recently published paper (Sacco et al. 2017), where muscles from two groups of female CD‐1 mice were analyzed for this study. The first group (8 weeks of age; *n* = 6 for contractile function and *n* = 6 for enzyme activities) were obtained from Charles River Canada and assigned to the low radiation exposure group (26 cGy). The second group (8 weeks of age, obtained through in‐house breeding of 10‐week‐old females obtained from Charles River Canada; *n* = 9 for contractile function and *n* = 6 for enzyme activities) and assigned to the moderate radiation exposure group (46 cGy). All animals were housed in groups of four to five mice for 6 months at room temperature on a 12‐h:12‐h light:dark cycle at the Brock University Comparative Biosciences Facility. All animals had access to a modified AIN‐93G diet (purchased from Harlan Laboratories) that contained vitamin‐free casein as the protein source (whole diet contains 19% of kcal from protein, 65% from carbohydrate, and 16% from fat) and water ad libitum. All experimental protocols were approved by the Brock University (St. Catharines) Animal Care Committee and conformed to the guidelines of the Canadian Council on Animal Care.

### Scanning protocol

The right hind limb of each mouse was scanned in vivo with *μ*CT (Skyscan 1176, Bruker microCT, Kontich, Belgium) at three time points, with each time point separated by 2 months as previously reported (Sacco et al. 2017). The left hind limb was out of the view of the X‐ray and was only scanned at the 6 months time period to serve as an internal nonradiated control (Sacco et al. 2017). The in vivo *μ*CT scanning parameters for the 26 cGy dose group were 9 *μ*m pixel size, 50 kV, 100 *μ*A, 1 mm Al filter, angular rotation step of 0.8^o^, and an exposure time of 3300 msec, resulting in a total scan time of 15 min 11 sec (Sacco et al. 2017). The parameters for the 46 cGy dose group were 9 *μ*m pixel size, 40 kV, 300 *μ*A, 1 mm Al filter, angular rotation step of 0.8°, and an exposure time of 3350 msec, resulting in a total scan time of 16 min 23 sec (Sacco et al. 2017). All scans were performed over 180° with no frame averaging.

### Muscle collection

All mice experienced an overnight fast before any surgical procedures were carried out. Mice were anesthetized in an induction chamber using isoflurane (4–5%) dissolved in oxygen, transitioned to a nose cone for maintenance of the anesthesia (2% delivery rate) (Sacco et al. 2017), and placed on the surgical bed for muscles (soleus and extensor digitorum longus [EDL]) to be surgically removed. Six mice from the low‐dose radiation group and nine from the moderate dose were used for in vitro muscle contraction. Six more mice from both radiation doses were used for enzyme activity analysis. Following muscle collection, mice were euthanized by an overdose of sodium pentobarbital (120 mg/kg body weight).

### In vitro isometric muscle function

Soleus and EDL were dissected from distal to proximal tendon after being secured with braided silk sutures, and were immediately mounted in an in vitro skeletal muscle testing system (Model 1200a; Aurora Scientific Inc.) for contractile measures as previously reported (Gittings et al. 2012). Data acquisition was performed using a Model 600a software, version 1.60 (Aurora Scientific Inc.) and muscle stimulation via flanking platinum electrodes driven by a Model 701B Bi‐phase stimulator (Aurora Scientific Inc.). Muscles were suspended in a jacketed organ bath‐containing oxygenated (95% O_2_ and 5% CO_2_) Tyrode's solution (121 NaCl, 5 KCl, 24 NaHCO_3_, 0.4 NaH_2_PO_4_, 0.5 MgCl_2_, 1.8 CaCl_2_, 5.5 D‐glucose, and 0.1 EDTA, pH 7.4) maintained at 25.0 ± 0.1°C (Lannergren et al. 2000), and left to equilibrate for 30 min.

Following the equilibration period, supramaximal stimulus intensity was determined by eliciting a twitch contraction every ~10 sec with increasing current until maximum twitch force was observed to ensure all fibers in the preparation were activated (voltage ≥ 60 V). Stimulation was set to ~1.25 the intensity required for peak twitch force for the remainder of the experiment. To determine the optimal length for maximal twitch force (*L*_o_), muscles resting at just‐taut length were stimulated every ~10 sec as muscle length was increased in small increments until peak active force was observed. Muscle length at *L*_o_ was determined, using a horizontal zoom stereoscope for EDL muscles and digital Vernier caliper for soleus muscles (experiments for each muscle type occurred simultaneously on identical systems).

After preliminary procedures, the maximum isometric twitch and tetanic forces were obtained at *L*_o_. Twitch force (*P*_t_) was measured as the maximum force produced in response to a single stimulation (i.e., 1 Hz, 0.1 msec pulse width). Otherwise, peak tetanic force (*P*_o_) was determined by brief (500 msec) supramaximal stimulation frequency (150 Hz for EDL and 100 Hz for soleus). From these values, the twitch:tetanus ratio (*P*_t_:*P*_o_) was calculated. In subsequent analysis, the time to peak tension (the elapsed time from the onset to the peak of isometric force development, TPT) and the half‐relaxation time (elapsed time from the peak to 50% of isometric force development, " RT) of twitch and tetanic force records were calculated. To measure the rate of force development (+d*P*/d*t*) and relaxation (−d*P*/d*t*), a smoothed graph showing the first derivative of the force‐time curve was plotted. From that, the maximal slope of the function during the rise and fall in force were used as rates of force development and relaxation, respectively. Following the collection of contractile measures, the muscles were removed from the bath and the sutures were cut off immediately distal to the muscle–tendon junctions. Muscles were briefly blotted dry to remove excess liquid, and mass was determined on a standard mass balance. The calculation for physiological cross‐sectional area was done as previously reported, using the optimal length and mass of each muscle (Grange et al. 2002).

### Enzyme activity

Soleus and EDL muscles from both hind limbs were removed, blotted on kimwipes to remove excess blood, snap frozen with liquid nitrogen, and stored in a −80°C freezer for later use. On the day of enzyme analysis, muscles were removed from the freezer and chipped under liquid nitrogen. Muscle chips (~12 mg) were suspended in 20 volumes (w/v) of 50 mM Tris‐HCl buffer (pH 7.8) and homogenized in a prechilled 2 mL glass homogenizer. Enzymes representative of amino acid metabolism (alanine aminotransferase), fatty acid metabolism (*β*‐Hydroxyacyl‐CoA dehydrogenase; carnitine palmitoyltransferase), glycolysis (hexokinase), and oxidative metabolism (citrate synthase; cytochrome c oxidase) were measured. All enzyme assays were measured using an Ultraspec 2100 Pro spectrophotometer (Biochrom, Cambridge, UK) at room temperature (25°C).

#### Alanine aminotransferase activity

Alanine aminotransferase (AAT; EC 2.6.1.2) activity was determined in muscle homogenate as previously described (Mole et al. 1973) with the following modifications. Reaction buffer included 75 mmol/L Tris‐HCl (pH 8.3), 5 mmol/L 2‐oxoglutarate, 0.18 mmol/L reduced nicotinamide adenine dinucleotide (NADH), 50 ug lactate dehydrogenase, and 200 mmol/L L‐alanine.

#### β‐Hydroxyacyl‐CoA dehydrogenase activity

*β*‐Hydroxyacyl‐CoA dehydrogenase (*β*‐HAD; EC 1.1.1.35) activity was determined in muscle homogenate as previously described (Bass et al. 1969) with the following modifications. Reaction buffer included 103.2 mmol/L triethanolamine‐HCl (pH 7.0), 5 mmol/L ethylenediaminetetraacetic acid (EDTA), 0.225 mmol/L NADH, and 0.1 mmol/L acetoacetyl‐CoA.

#### Carnitine palmitoyltransferase activity

Carnitine palmitoyltransferase (CPT; EC 2.3.1.21) activity was determined in muscle homogenate as previously described (Alhomida 2001) with the following modifications. Reaction buffer included 76.6 mmol/L Tris‐HCl (pH 8.0), 1.1 mmol/L EDTA, 0.24 mmol/L 5,5′‐dithio‐bis(2‐nitrobenzoic acid)(DTNB), 0.08 mmol/L palmitoyl‐CoA, and 1.1 mmol/L L‐carnitine. The homogenate was subjected to three freeze‐thaw cycles to ensure mitochondrial membrane rupture.

#### Hexokinase activity

Hexokinase (HK; EC 2.7.1.1) activity was determined in muscle homogenate as previously described (Thompson and Cooney 2000) with the following modifications. Reaction buffer included 40 mmol/L triethanolamine‐HCl (pH 7.6), 5 mmol/L EDTA, 7.5 mmol/L MgCl_2_, 0.42 mmol/L nicotinamide adenine dinucleotide phosphate (NADP), 2.5 mmol/L adenosine triphosphate (ATP), 10 *μ*g glucose‐6‐phosphate dehydrogenase, and 2.22 mmol/L D(+)‐glucose.

#### Citrate synthase activity

Citrate synthase (CS; EC 2.3.3.1) activity was determined in muscle homogenate as previously described (Srere 1969) with the following modifications. Reaction buffer included 60 mmol/L Tris‐base (pH 7.0); 0.1 mmol/L DTNB, 0.5 mmol/L acetyl‐CoA, 0.5% (v/v) Triton X‐100, and 0.5 mmol/L oxaloacetate. The homogenate was subjected to three freeze‐thaw cycles to ensure mitochondrial membrane rupture.

#### Cytochrome c oxidase activity

Cytochrome c oxidase (COX; EC 1.9.3.1) activity was determined in muscle homogenate as previously described (Cooperstein and Lazarow 1951) with the following modifications. Reaction buffer included 18.3 mmol/L phosphate buffer (pH 7.4), 3% (v/v) Tween 20, and 0.04 mmol/L cytochrome c. The homogenate was subjected to three freeze‐thaw cycles to ensure mitochondrial membrane rupture.

### Statistical analyses

All values are expressed as the means ± standard error (SEM). A paired two‐tailed *t*‐test (IBM SPSS) was used to establish significant differences (*P *<* *0.05) between control and irradiated muscles within each dose with respect to muscle function. A ratio paired two‐tailed *t*‐test (IBM SPSS) was used to establish significant differences (*P *<* *0.05) in fold change maximal enzyme activity between control and irradiated muscles within each dose. Outliers were determined and removed using the Grubbs' test for outliers (Grubbs 1969).

## Results

### Anthropometric muscle characterization

EDL and soleus mass, length, and CSA did not significantly differ between control and radiation groups for both low and moderate radiation doses (Table 1).

**Table 1:** Anthropometric characterization of mice extensor digitorum longus and soleus muscles with and without exposure to radiation doses by microcomputed tomography scanning

	EDL		SOL	
	CON	RAD	CON	RAD
Low dose (26 cGy)				
Mass (mg)	13 ± 0.2	14 ± 0.2	12 ± 0.2	12 ± 0.2
Length (mm)	13 ± 0.2	13 ± 0.0	12 ± 0.2	12 ± 0.1
CSA (mm^2^)	2.2 ± 0.1	2.3 ± 0.1	1.3 ± 0.1	1.4 ± 0.1
Moderate dose (46 cGy)				
Mass (mg)	12 ± 0.4	13 ± 0.5	12 ± 0.6	12 ± 0.4
Length (mm)	14 ± 0.2	14 ± 0.3	13 ± 0.2	13 ± 0.1
CSA (mm^2^)	1.9 ± 0.1	2.0 ± 0.1	1.2 ± 0.1	1.2 ± 0.1

Values are mean ± SEM. *n *=* *6 for low dose and *n *=* *9 for moderate dose. CON, no radiation exposure; RAD, radiation exposure; SOL, soleus; EDL, extensor digitorum longus; CSA, cross‐sectional area.

### Isometric muscle function

**Figure 1:**
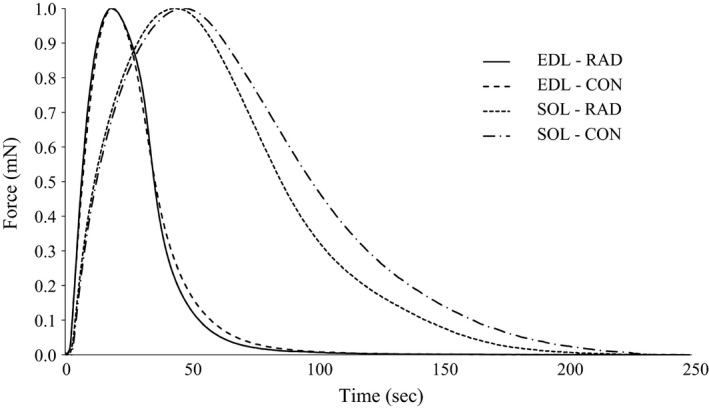
Representative peak twitch force trace normalized to maximal peak twitch of mice extensor digitorum longus and soleus muscles with and without exposure to the low (26 cGy) radiation by microcomputed tomography scanning. EDL, extensor digitorum longus; SOL, soleus; CON, no radiation exposure; RAD, radiation exposure.

**Figure 2:**
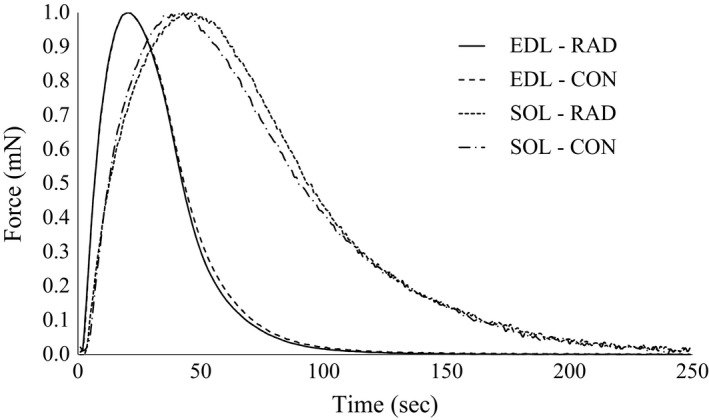
Representative peak twitch force trace normalized to maximal peak twitch of mice extensor digitorum longus and soleus muscles with and without exposure to moderate (46 cGy) radiation by microcomputed tomography scanning. EDL, extensor digitorum longus; SOL, soleus; CON, no radiation exposure; RAD, radiation exposure.

Given the small diameter of the muscles and the lower temperature used in this study, it is unlikely that muscle viability was compromised by inadequate oxygen or substrate delivery during the experiments (Barclay 2005). Representative twitch force traces for the low and moderate radiation doses are presented in Figure 1 and 2, respectively. The isometric functional characteristics for the EDL and soleus did not differ between groups within the low‐radiation dose (Table 2). In contrast, there was a 5% increased time‐to‐peak tension and a 16% increased half‐relaxation time in EDL in the moderate radiation dose group compared to control, however these differences were not seen when standardized to peak twitch force (Table 3).

**Table 2:** Isometric muscle function of mice extensor digitorum longus and soleus muscles with and without exposure to low (26 cGy) radiation by microcomputed tomography scanning

	EDL		SOL	
	CON	RAD	CON	RAD
*P*_t_ (mN)	52 ± 6	70 ± 7	25 ± 2	24 ± 1
*P*_o_ (mN)	350 ± 31	406 ± 18	229 ± 22	228 ± 11
*P*_t_/CSA (mN/mm^2^)	23 ± 2	31 ± 3	19 ± 2	18 ± 1
*P*_o_/CSA (mN/mm^2^)	156 ± 13	181 ± 8	172 ± 15	168 ± 10
*P*_t_:*P*_o_	0.15 ± 0.01	0.17 ± 0.01	0.11 ± 0.01	0.11 ± 0.01
TPT(msec)	17 ± 0.3	17 ± 0.3	43.0 ± 1.5	40.0 ± 1.5
1/2 RT(msec)	17 ± 0.5	17 ± 0.8	42.3 ± 2.3	40.8 ± 3.1
TPT/*P*_t_ (msec/mN)	0.4 ± 0.1	0.3 ± 0.0	1.8 ± 0.2	1.4 ± 0.3
1/2 RT/*P*_t_ (msec/mN)	0.4 ± 0.1	0.3 ± 0.0	1.8 ± 0.2	1.4 ± 0.3
+d*P*/d*t* (mN/msec)	3.2 ± 0.4	4.3 ± 0.4	1.1 ± 0.1	1.0 ± 0.1
−d*P*/d*t* (mN/msec)	1.9 ± 0.2	2.7 ± 0.3	0.4 ± 0.0	0.4 ± 0.0

Values are mean ± SEM. *n *=* *6. EDL, extensor digitorum longus; SOL, soleus; CON, no radiation exposure; RAD, radiation exposure; *P*_t_, peak twitch force; *P*_o_, peak tetanic force; CSA, cross‐sectional area; *P*_t_:*P*_o_, twitch to tetanus ratio; TPT, time‐to‐peak tension; 1/2 RT, half‐relaxation time; +d*P*/d*t*, rate of force development; −d*P*/d*t*, rate of force relaxation.

**Table 3:** Isometric muscle function of mice extensor digitorum longus and soleus muscles with and without exposure to moderate (46 cGy) radiation by microcomputed tomography scanning

	EDL		SOL	
	CON	RAD	CON	RAD
*P*_t_ (mN)	74 ± 4	77 ± 6	33 ± 2	30 ± 2
*P*_o_ (mN)	388 ± 23	394 ± 16	228 ± 18	213 ± 15
*P*_t_/CSA (mN/mm^2^)	39 ± 1	40 ± 2	28 ± 3	26 ± 1
*P*_o_/CSA (mN/mm^2^)	202 ± 8	206 ± 5	189 ± 15	188 ± 15
*P*_t_:*P*_o_	0.19 ± 0.01	0.19 ± 0.01	0.15 ± 0.01	0.15 ± 0.01
TPT (msec)	18 ± 0.3	19 ± 0.3^a^	42 ± 2	45 ± 2
1/2 RT (msec)	24 ± 1	28 ± 1^a^	52 ± 4	54 ± 3
TPT/*P*_t_ (msec/mN)	0.3 ± 0.0	0.3 ± 0.0	1.3 ± 0.1	1.6 ± 0.1
1/2 RT/P_t_ (msec/mN)	0.3 ± 0.0	0.4 ± 0.0	1.6 ± 0.2	1.9 ± 0.2
+d*P*/d*t* (mN/msec)	6.4 ± 0.4	6.6 ± 0.5	1.7 ± 0.1	1.6 ± 0.1
−d*P*/d*t* (mN/msec)	2.4 ± 0.2	2.4 ± 0.2	0.5 ± 0.0	0.4 ± 0.0

Values are mean ± SEM. *n *=* *9. EDL, extensor digitorum longus; SOL, soleus; CON, no radiation exposure; RAD, radiation exposure; *P*_t_, peak twitch force; *P*_o_, peak tetanic force; CSA, cross‐sectional area; *P*_t_:*P*_o_, twitch to tetanus ratio; TPT, time‐to‐peak tension; 1/2 RT, half‐relaxation time; +d*P*/d*t*, rate of force development; −d*P*/d*t*, rate of force relaxation.

a Significantly different from CON.

### Enzyme activity

**Figure 3:**
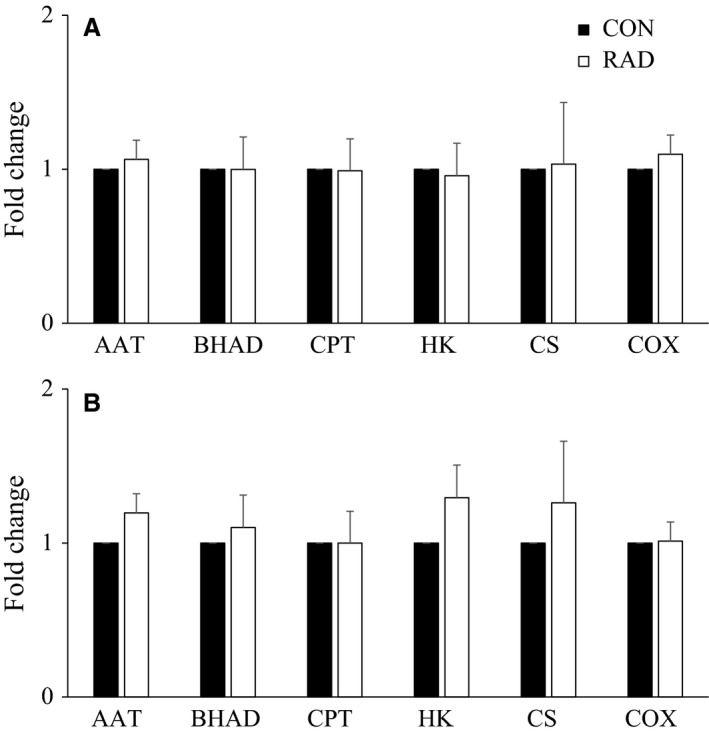
Fold change enzyme activity between radiated and nonradiated mice (A) extensor digitorum longus (EDL) and (B) soleus muscles exposed to low (26 cGy) radiation by microcomputed tomography scanning. Values are mean ± SEM. *n *=* *5–6. CON, no radiation exposure; RAD, radiation exposure; AAT, alanine aminotransferase; BHAD, 3‐hydroxyacyl‐CoA dehydrogenase; CPT, carnitine palmitoyltransferase; HK, hexokinase; CS, citrate synthase; COX, cytochrome c oxidase.

**Figure 4:**
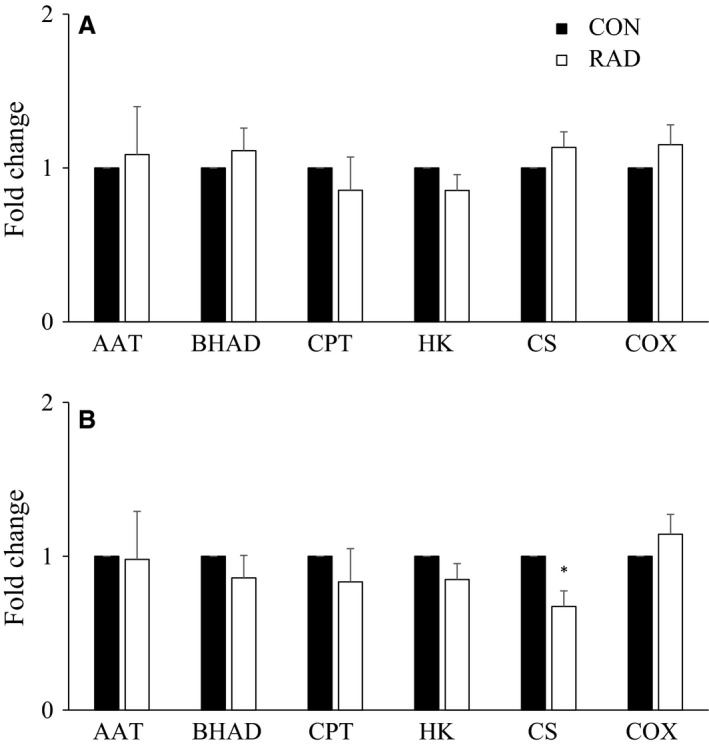
Fold change enzyme activity between radiated and nonradiated mice (A) extensor digitorum longus (EDL) and (B) soleus muscles exposed to moderate (46 cGy) radiation by microcomputed tomography scanning. Values are mean ± SEM. *n *=* *5–6. *Significantly different from CON. CON, no radiation exposure; RAD, radiation exposure; AAT, alanine aminotransferase; BHAD, 3‐hydroxyacyl‐CoA dehydrogenase; CPT, carnitine palmitoyltransferase; HK, hexokinase; CS, citrate synthase; COX, cytochrome c oxidase.

Fold changes of maximal enzyme activities in soleus and EDL did not differ significantly between control and low (Fig. 3), or moderate (Fig. 4) dose irradiation. The only exception was a ~33% decrease in CS activity in moderate dose‐irradiated soleus compared to control.

## Discussion

When analyzing bone structure using *μ*CT, the exposure to radiation of skeletal muscle neighboring bone is unavoidable. It is important, therefore, to assess the effects of this exposure on muscle contractile function and metabolism. This study is the first to measure the effects of repeated in vivo *μ*CT radiation on mouse EDL and soleus in vitro contractile function and metabolic activity. Our findings demonstrate that exposure to low dose radiation had no effect on skeletal muscle contractile function, and maximal enzyme activity. In contrast, exposure to moderate dose radiation resulted in lower soleus maximal citrate synthase activity. This suggests that studies that examine both tibial bone microarchitecture by *μ*CT and its adjacent muscle unit should be aware of radiation exposure dose when establishing scanning parameters for longitudinal repeated measures.

### Muscle morphometrics, contractile function, and metabolic activity is not affected by low‐dose radiation

To the authors' knowledge, this is the first study to examine the impact of low‐dose X‐ray radiation on rodent skeletal muscle morphometrics, contractile function, and metabolic activity. Repeated exposure (every 2 months) to low‐dose radiation (26 cGy) over a 6‐month period had no impact on EDL and soleus length, cross sectional area, muscle contractile function, or metabolic activity compared to control. Given the novelty of this study, comparison to previous research is difficult as these studies examined X‐ray doses 2–60 times higher than the low dose used in this study (Gubina et al. 2010; Hardee et al. 2014; Chowdhury et al. 2016). However, some studies have examined low‐dose radiation (<30 cGy) exposure on rodent skeletal muscle, using sources other than X‐rays, including ^56^Fe (Bandstra et al. 2009; Shtifman et al. 2013) and *γ* (Masuda et al. 2015) radiation. Low‐dose (5–25 cGy) whole body *γ* radiation daily for 30 consecutive days caused a decrease in myonuclei and satellite cell number per myofiber in EDL, which was not seen 3 months postexposure (Masuda et al. 2015). Similarly, a single low‐dose (15–31 cGy) whole body ^56^Fe radiation caused increased centralized nuclei (surrogate measure of muscle regeneration) and decreased fiber cross‐sectional area of triceps brachii 9 weeks postexposure (Bandstra et al. 2009) and flexor digitorum brevis 7 days postexposure (Shtifman et al. 2013). Based on these studies, it appears that the impact of low‐dose radiation on skeletal muscle may be dependent on the source and the effects are somewhat acute and dissipate after 2–3 months. Although not measured in this study, it is possible that localized low‐dose X‐ray radiation may influence skeletal muscle turnover similar to other forms of radiation administered at the whole‐body level, but this did not translate into changes in skeletal muscle weight, cross‐sectional area, contractile function, or metabolic activity. Future studies should examine if repeated localized exposure to low‐dose X‐ray radiation causes increased skeletal muscle turnover. Also, given that the control hind limb was subject to a single dose of radiation at 6 months, and could potentially underestimate the effects of *μ*CT on muscle contraction and maximal enzyme activity, future studies could address this by comparing the irradiated hind limb with that of a zero‐dose control hind limb.

### Moderate radiation exposure minimally affects muscle contractile function and decreases CS activity

Repeated exposure to a moderate dose of X‐ray radiation had no impact on EDL and soleus mass, length, or cross sectional area. As previously stated, few studies have examined X‐ray radiation exposure, most in the high to very high dose range. However, one study examined a single moderate (50 cGy) X‐ray dose isolated to a mouse hind limb, demonstrating increased cellular signals associated with protein synthesis and degradation 2 days postexposure (Fix et al. 2016). Thus, it would appear that similar to low‐dose, moderate‐dose radiation exposure influences skeletal muscle turnover acutely but not chronically.

Exposure to a moderate dose of radiation resulted in a 5% increase in time‐to‐peak tension and a 16% increase in half‐relaxation time in EDL compared to control. Although time‐to‐peak tension and half‐relaxation time lengthened with irradiation, the maximal rates of force development (+d*P*/d*t*) and relaxation (−d*P*/d*t*) were not significantly different between irradiated and control EDL. It is generally well accepted that parameters of force kinetics (e.g. time‐to‐peak twitch and half‐relaxation time) are dependent on force amplitude. Although not significant, EDL peak twitch force did increase with moderate radiation exposure and when time‐to‐peak twitch and half‐relaxation time were normalized to peak twitch force, twitch kinetics between CON and RAD EDL were not significantly different.

The activity of citrate synthase, a marker of mitochondrial oxidative capacity, in soleus muscles that were irradiated at the moderate dose was ~33% lower compared to control. This contrasts with no change in cytochrome c oxidase, a member of the electron transport chain and an alternative marker of mitochondrial oxidative capacity. The susceptibility of citrate synthase to a moderate dose of radiation, with no change in members of the electron transport chains, has been previously demonstrated in gill and skin of rainbow trout. A single 10–50 cGy dose of *γ* radiation resulted in a 50–75% decrease in citrate synthase activity in both gill and skin in vitro 2 h postexposure with no change in spleen or in the activities of complexes *I*–*V* of the electron transport chain (O'Dowd et al. 2009). Based on this study and previous research, highly oxidative tissues (e.g. mouse soleus, fish gills, and skin) which contain more mitochondria are more sensitive to the effects of a moderate dose of radiation compared to less oxidative tissues (e.g. mouse EDL, fish spleen) which contain less mitochondria. Despite this decrease in citrate synthase activity in irradiated soleus, this did not influence contractile function or maximal activities of other metabolic enzymes. Future research should examine isolated myofiber bioenergetics to determine if this change in citrate synthase maximal activity influences cellular respiration.

### Summary

Overall, we conclude that exposure to 46 cGy, but not 26 cGy, has an impact on maximal citrate synthase activity of soleus, but not EDL, in female mice. EDL exhibited an increase in time‐to‐peak tension and half relaxation time after exposure to the moderate dose, which was lost when normalized to absolute force. Moderate dose (i.e. 46 cGy) compared to low‐dose (i.e. 26 cGy) radiation has improved image resolution and quality, but both doses provided reasonable image contrast when examining bone microarchitecture (Sacco et al. 2017). Thus, longitudinal *μ*CT scanning at a low dose will provide sufficient quality bone images (Laperre et al. 2011; Sacco et al. 2017) and not impact contractile function or metabolic profile of neighboring muscles. This allows more researchers to begin to develop longitudinal scanning protocols as they allow the use of fewer animals and can show the effect of an intervention over time in the same animal.

## Conflicts of Interest

The authors declare that there are no conflicts of interest in regard to the publication of this paper.
